# Guanylate cyclase activity in moss: revisiting the role of ERECTA-like receptors

**DOI:** 10.1007/s12298-025-01606-1

**Published:** 2025-06-04

**Authors:** Klaudia Hammer, Brygida Świeżawska-Boniecka, Mateusz Kwiatkowski, Benedetta Cencini, Adriana Szmidt-Jaworska, Krzysztof Jaworski

**Affiliations:** 1 Department of Plant Physiology and Biotechnology, Nicolaus Copernicus University, Torun, Poland; 2 Department of Chemistry, Biology and Biotechnology, Università Degli Studi Di Perugia, Piazza Universita 1, 06123 Perugia, Italy

**Keywords:** Guanylate cyclase (GC), Calcium ions, LRR-RLK, Kinase, *Physcomitrium patens*

## Abstract

The structural complexity of plant proteins, particularly receptor-like kinases, has garnered significant attention in recent research. This research identifies *Physcomitrium patens* ERECTA-like receptor 1 (PpERL1) as a new guanylate cyclase (GC) within the cytoplasmic kinase domain by examining its structural and functional properties. Comprehensive sequence alignment analyses reveal substantial variability among ERECTA-like proteins from mosses in contrast to vascular plants, while GC motifs display remarkable conservation, suggesting a critical functional relevance. In vitro tests validate the GC activity of recombinant PpERL1, with key residue substitutions at positions 1 and 14 leading to a decrease in GC activity. Notably, cGMP does not impact PpERL1's kinase activity, while inhibits its enzymatic function, contrasting with regulatory mechanisms observed in vascular plant GCs. Independent regulatory mechanisms are shown by calcium ions increasing GC activity without affecting kinase functioning. These results demonstrate an evolutionary divergence in the regulatory interactions between GC and kinase domains in mosses versus vascular plants, reflecting adaptive strategies unique to non-vascular plant lineages.

**Supplementary Information:**

The online version contains supplementary material available at 10.1007/s12298-025-01606-1.

## Introduction

Accumulating evidence underscores the crucial role of cyclic guanosine-3′,5′-monophosphate (cGMP) as a signaling molecule in diverse plant signal transduction pathways. Recent years have seen an increase in reports identifying plant guanylate cyclases (GCs), the enzymes responsible for cGMP synthesis, as central components in various physiological processes (Gehring and Turek 4). Current research implicates plant GCs in brassinosteroid-mediated signaling (Kwezi et al. 10), water and ionic homeostasis (Turek and Gehring 26), photoperiodism (Szmidt-Jaworska et al. 24), pollen tube reorientation (Vaz Dias et al. 28; Wong et al. 31), growth and differentiation regulation (Kwezi et al. 11), and responses to tissue damage and immunity (Świeżawska et al. 21, 22; Malukani et al. 14; Duszyn et al. 3). Plant GCs are typically identified by a conserved 14-amino-acid motif, [KS]x[SCG]x{10}[KR]x{0,3}[DHSE], featuring residues essential to the catalytic function. This motif was derived by aligning known catalytic centers of bacterial and animal GCs (Ludidi and Gehring 13; Wong et al. 30).

Genome-wide analyses of plant genomes have revealed GC activity primarily within receptor-like proteins, including AtBRI1 (Kwezi et al. 10), AtPSKR1 (Kwezi et al. 11), AtWAKL10 (Meier et al. 15), AtPNP-R1 (Turek and Gehring 26), HpPepR1 (Świeżawska et al. 22), and BdERL1 (Świeżawska-Boniecka et al. 23). These receptors belong to a class of “moonlighting” proteins, which possess multi-domain architectures that support various biochemical functions (Turek and Irving 27). Structurally, they comprise an extracellular N-terminal receptor domain and an intracellular cytosolic kinase domain with an embedded GC catalytic motif. The GC domain organization in these plant proteins shows architectural parallels to animal transmembrane GCs (tmGCs); however, in animal tmGCs, the GC domain is situated at the C-terminus, distal to the kinase domain (Irving et al. 6). In plant GCs, nearly all identified examples are associated with leucine-rich repeats (LRRs) in the extracellular domain, classifying them as LRR-RLKs (Leucine-Rich Repeat Receptor-Like Kinases) (Torii 25). This includes the recently characterized BdERL1 (ERECTA-like 1) from *Brachypodium distachyon* (Turek and Irving 27). Remarkably, the GC catalytic center sequence shows high conservation across these plant proteins, underscoring evolutionary significance. However, all identified GC domains to date are restricted to vascular plant species, including *Arabidopsis thaliana*, *Brachypodium distachyon*, *Hippeastrum x hybridum*, *Zea mays*, *Oryza sativa*, and *Pharbitis nil*.

In contrast, evidence for GCs in non-vascular plants remains lacking (Turek and Irving 27). Only limited reports have confirmed the presence of any cyclases in bryophytes, such as the identification of *Marchantia polymorpha* MpCAPE (Kasahara et al. 8) and TIR1/AFB orthologs in *Physcomitrium patens* as adenylate cyclases (AC) (Qi et al. 19). Our genome analysis of *Physcomitrium patens* identified a GC catalytic center motif within the LRR receptor-like serine/threonine-protein kinase ERL1 isoform X1. Members of the ERECTA (ERf) protein family, including ERECTA (ER) along with its paralogs ERECTA-LIKE 1 (ERL1) and ERECTA-LIKE 2 (ERL2), bind EPF (Epidermal Patterning Factor)/EPFL (EPF-like) signal peptides, playing a key role in inflorescence development and epidermal organization (Shpak 20). To date, GC functionality within the ERECTA protein family has been confirmed exclusively in *Brachypodium distachyon* (Świeżawska-Boniecka et al. 23).

This report aims to validate the first observed instance of GC activity in non-vascular plants, specifically within the ERECTA-like 1 receptor protein (PpERL1) of *Physcomitrium patens*. Furthermore, it examines the potential regulatory impact of this GC activity on the protein's intrinsic kinase domain, underscoring the evolutionary conservation of the GC catalytic motif and receptor kinase domain organization across diverse plant taxa.

## Materials and methods

### Bioinformatic analysis

The presence of GC domain in PpERL1 was verified using the ScanProsite tool with the following GC motif: [KS]x[SCG]x{10}[KR]x{0,3}[DHSE]. The calmodulin-binding site was detected with Calmodulin Target Database (http://calcium.uhnres.utoronto.ca/ctdb/ctdb/sequence.html). The prediction of the EF-hand motif was performed with CAL-EF-AFi server (https://14.139.62.220/calb/index.html).Protein sequence comparisons were conducted using the Basic Local Alignment Search Tool (BLAST) with the BLOSUM62 matrix and standard gap costs (existence: 11; extension: 1). Phylogenetic trees were generated using PhyML Maximum likelihood method with 100 bootstrap replicates. The presence of cGMP-binding motifs in PpERL1, BdERL1 (XP_003561134.3), BdPepR2 (XP_003571653), and AtBRI1 (NP_195650.1) was assessed using the ScanProsite tool with accession number PS00889.

### Construction of the expression vector, expression and purification of recombinant protein

The analyzed fragment of *Physcomitrium patens* ERECTA-like 1 (NCBI accession number: XP_024359500.1) encodes the intracellular kinase domain, containing the conserved GC motif within the 658–1018 amino acid region, specifically located at positions 877–893. The cDNA of *Physcomitrium patens* PpERL1 was amplified via PCR using specific primers (Supplementary Table S1) according to the CloneAmp™ HiFi PCR Premix Protocol-At-A-Glance (Takara Bio USA, Inc.). The obtained insert was cloned into the linearized expression vector pGEX-6P-2 at the *EcoRI* and *XhoI* restriction sites using the In-Fusion® HD EcoDry™ Cloning Plus kit (Takara Bio USA, Inc.). The correctness of the insert was verified through sequencing. The resulting plasmid was introduced into the expression strain *E. coli* BL21(DE3)pLysS (Promega) using the heat shock method (42 °C for 20 s). A positively transformed colony was used to overexpress GST-tagged recombinant proteins. Transformed cells were cultured in LB medium supplemented with 2% glucose at 37 °C and 100 mg/mL ampicillin with agitation. Fusion protein production was induced at an optical density (OD_600_) of 0.6 by adding IPTG (isopropyl β-D-thiogalactoside) to a final concentration of 0.1 mM, followed by incubation at 18 °C for 18 h. The bacterial cells were collected by centrifugation, and the obtained pellet was suspended in lysis buffer (50 mM Tris–HCl (pH 8.0), 150 mM NaCl, 5 mM EDTA, 5 mM EGTA, 1 mM PMSF, a protease inhibitor cocktail, and 0.2 mg/mL lysozyme) at a ratio of 1 g of pellet to 10 mL of buffer. The suspension was incubated for 10 min at room temperature and then for 20 min on ice. Cell membranes were disrupted using sonication (4 pulses of 15 s each). The lysate was then supplemented with 1 mM DTT, 1% Triton X-100, and 1 mM PMSF, incubated for 10 min on ice, and centrifuged at 4 °C for 20 min at 12,500 × g. The soluble fraction was adsorbed onto glutathione-Sepharose 4B resin and washed with 1 × phosphate-buffered saline (PBS). Recombinant proteins were eluted with 10 mM glutathione in 50 mM Tris–HCl (pH 9.0). The purity and homogeneity of the obtained fractions of fusion proteins were analyzed by 8.5% (v/v) SDS-PAGE stained with Coomassie blue.

### Site-directed mutagenesis

The confirmation of functionality of the predicted GC domain in the PpERL1 protein, was conducted by targeted mutagenesis on two functionally significant amino acids within the GC catalytic center motif. Specifically, the serine (S877) at position 1 was substituted with glycine (G) to create the S877G mutant. Additionally, the lysine (K890) at position 14 was replaced with asparagine (N), resulting in the K890 N mutant. Site-directed mutagenesis was performed using the QuikChange II XL Site-Directed Mutagenesis Kit (Agilent) according to the manufacturer's protocol. PCR amplification was conducted with specific primers (Supplementary Table S2) designed using the QuikChange® Primer Design Program (https://www.agilent.com/store/primerDesignProgram.jsp) and a previously constructed plasmid, pGEX-6P-2, as the DNA template. Following amplification, the methylated template plasmid was digested with the restriction enzyme *Dpn*I at 37 °C for 1 h to eliminate the parental DNA. The resulting plasmids containing the mutated fragments of the *PpERL1* gene were then cloned into competent *E. coli* XL10-Gold cells (Agilent) using the heat shock method at 42 °C for 30 s. Transformants were inoculated onto LB agar plates supplemented with ampicillin (100 mg/mL) and incubated overnight at 37 °C. The accuracy of the mutations was confirmed through sequencing.

### Determination of guanylate cyclase domain activity

The GC activity of GST-tagged recombinant proteins was assessed by quantifying the amount of cGMP produced in the in vitro enzymatic reactions. Each reaction mixture, with a final volume of 100 µL, contained 5 µg of fusion protein, 1 mM DTT, 50 mM Tris–HCl (pH 7.5), 5 mM MgCl_2_, 2.5 mM MnCl_2_, and GTP at varying concentrations of 0.1–3 mM for the wild-type and 1 mM for the mutants. An additional variant of the PpERL1 wild-type was supplemented with 0.25 mM CaCl_2_. Reactions were conducted for 20 min at 30 °C and terminated by heating at 100 °C for 5 min to denature the proteins. The samples were then centrifuged at 12,000 × g for 15 min. Negative controls included reactions without substrate and reactions without enzyme for each GTP concentration tested. The amount of cGMP produced was measured in 5 µL of the sample using LC–MS/MS. The quantification of cGMP content was based on standard curves that ranged from 0 to 200 pg (0–0.7 pmol). LC–MS/MS experiments were conducted by Nexera UHPLC and LCMS-8045 integrated system (Shimadzu Corporation). The analyses were performed in negative ESI mode, using pure cGMP standard dissolved in HPLC-grade water. Samples were separated on XSelect CSH C18 column (Waters, Ireland) using a gradient of B: 2%−10% in 8.5 min (solvent A: water with 0.1% formic acid v/v; solvent B: acetonitrile with 0.05% formic acid v/v) at a flow rate of 0.3 ml/min and a voltage of 4.0 kV.

### Determination of kinase domain activity

The kinase activity of PpERL1 was evaluated using the Kinase-Glo® Plus Luminescent Kinase Assay kit (Promega), following the provided protocol. The reaction mixtures, with a final volume of 50 μL, included 2.5 μg of either the GST-PpERL1 wild-type or the GST-PpERL1 K890 N fusion protein, along with 25 mM Tris–HCl (pH 7.5), 5 mM MgCl_2_, 1 mM DTT, 5 μg/μL Histone Type III, and 10 μM ATP. Additionally, the mixtures contained optional components: 2 μM cGMP, 1 mM GTP, 1 mM GTP supplemented with 2 μM CaCl_2_, or 1 mM GTP with 2 μM CaCl_2_ and 1.2 μg of AtCaM7/AtCaM1. Reactions were performed at 30 °C for 15 min, stopped by adding 50 μl of Kinase-Glo® Reagent, and equilibrated at RT for 10 min. Luminescence was measured using a Synergy HT Multi-Mode Microplate Reader (BioTek). The enzymatic activity of the kinase domain was quantified as a relative value, expressed as a percentage, reflecting the inverse relationship between the amount of ATP consumed and the luminescence detected.

## Results and discussion

### Analysis of the PpERL1 amino acid sequence and evolutionary aspects

**Fig. 1:**
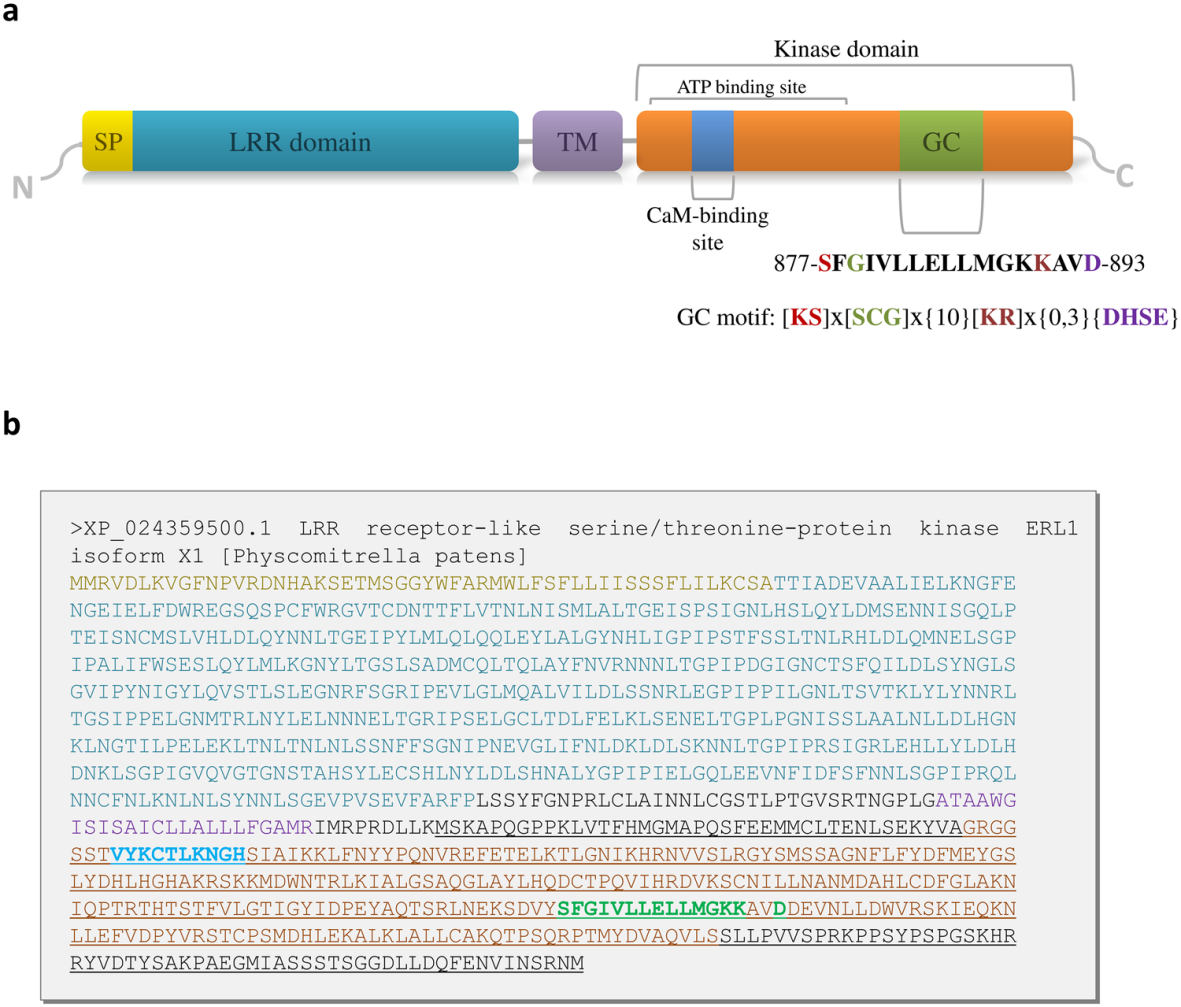
Sequence analysis of PpERL1 protein. **a** Schematic representation of the domain structure in transmembrane guanylate cyclases. Key domains are denoted as follows: TM – transmembrane domain; GC – catalytic guanylate cyclase domain. **b** Amino acid sequence of the PpERL1 protein from *Physcomitrium patens*. The extracellular domain is highlighted in turquoise, the transmembrane domain in purple, and the kinase domain in orange. Within the kinase domain, the GC catalytic motif is bolded and marked in green, with specific indication of the Mg^2^⁺/Mn^2^⁺ binding site. The CaM-binding site is also bolded and highlighted in blue. The analyzed protein fragment is underlined

The *Physcomitrium patens* ERECTA-like 1 gene (NCBI: LOC112274326) encodes a leucine-rich repeat (LRR) receptor-like serine/threonine-protein kinase. Its coding sequence (CDS) spans 3,057 bp, resulting in a 1,018-amino acid (aa) protein with an estimated molecular weight of 112.8 kDa. The protein structure includes an extracellular N-terminal hydrophobic signal peptide (1–52 aa) linked to the LRR domain (55–611aa) binding Epidermal Patterning Factors (EPF/EPF-like). This is followed by a transmembrane segment and an intracellular serine/threonine kinase domain (697–958 aa) that comprises both an ATP-binding site (697–834 aa) and a GC domain (877–893 aa). The GC motif within PpERL1, represented by the sequence [877-SFGIVLLELLMGKKAVD-893], includes conserved residues critical for catalytic function (Fig. 1a). Specifically, the serine at position 1 enables hydrogen bonding with guanine, glycine at position 3 contributes to GTP specificity, lysine at position 14 stabilizes cyclization, and aspartic acid at position 17 coordinates Mg^2^⁺ or Mn^2^⁺ cofactors (Wong et al. 30). Based on the structure and experimental assessment of BdERL1, AtPSKR1 and AtBRI1, we performed bioinformatic analysis, which revealed the presence of a calmodulin-binding motif (CaM) within the kinase domain (704-VYKCTLKNGH-713) that partially overlaps with the CaM-binding motif defined for BdERL1 (Fig. 1b), but not with those of AtBRI1 and AtPSKR1. CaM-binding site present in these two *A. thaliana* LRR-RLKs is detected in a homologous sequence of PpERL1 but with a lower score. In contrast, the homologous sequence of potential CaM-binding motif in ERL1 is not scored in AtBRI1 and AtPSKR1 (Oh et al. 17; Hartmann et al. 5; Świeżawska-Boniecka et al. 23). Additionally, no EF-hand motifs for Ca^2^⁺ binding were identified, consistent with findings for BdERL1 (Świeżawska-Boniecka et al. 23). Due to the established effect of cGMP on kinase activities in AtBRI1, BdPepR2, and BdERL1, we examined PpERL1 and these proteins for potential cNMP-binding sites. However, no hits were detected (Wheeler et al. 29; Świeżawska-Boniecka et al. 23; Duszyn et al. 2).

**Fig. 2:**
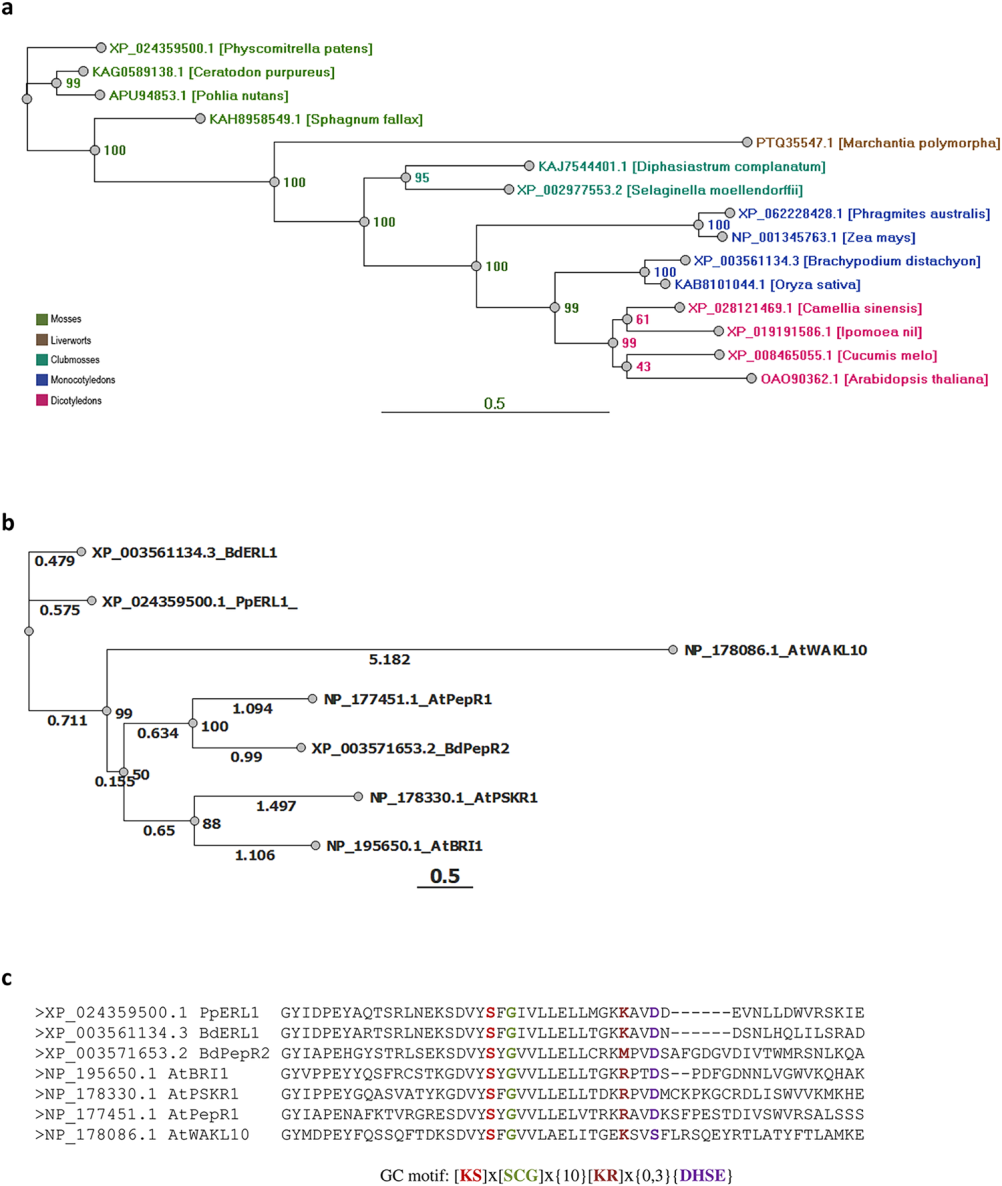
Evolutionary analysis and structural comparison of PpERL1 with LRR-RLK family members. **a** Phylogenetic tree depicting leucine-rich-repeat receptor-like kinase (LRR-RLK) family members across various clades with the highest homology to PpERL1. **b** Phylogenetic tree illustrating the evolutionary relationship between PpERL1 and selected representative LRR-RLKs. **c** Sequence alignment of PpERL1 with selected representative LRR-RLKs, focusing on the GC motif for comparative analysis

The ERECTA family in *P. patens* consists of 11 members, including PpERL1. These members are considered paralogs of each other. The amino acid sequence of *PpERL1* presents the highest sequence identity within the LRR-RLK family in mosses, with identities of 83.5% to *Ceratodon purpureus*, ~ 81% to *Pohlia nutans*, and 73.6% to *Sphagnum fallax*. Similarity decreases progressively with species from other plant lineages, including clubmosses such as *Selaginella moellendorffii* and *Diphasiastrum complanatum* (~ 57%), liverworts like *Marchantia polymorpha* (53%), monocots such as *Phragmites australis* (57%), *Zea mays* (56%), *Oryza sativa* (~ 55%), and *Brachypodium distachyon* (53%). Among eudicots, sequence identity is similarly moderate, with 56.4% identity to *Cucumis melo*, 55.6% to *Camellia sinensis*, 55.5% to *Arabidopsis thaliana*, and 55% to *Ipomoea nil*. Previous studies on *Brachypodium distachyon* ERECTA-like 1 show that ERf sequences exhibit over 90% similarity within monocots and above 70% between monocots and dicots, illustrating strong clade-specific conservation (Świeżawska-Boniecka et al. 23). Phylogenetic analysis of ERf sequences, along with the degree of sequence identity (Fig. 2a), reveals significant evolutionary divergence within the ERECTA-like protein family, particularly between mosses and vascular plants. Despite this divergence, the GC catalytic center motif remains highly conserved across the ERECTA family and other LRR-RLKs, including BRI1, PEPR1, PSKR1, and WAKL10 (Fig. 2b). Multiple sequence alignments of over 30 ERf homologs and select representative LRR-RLKs reveal complete conservation of serine at position 1 and glycine at position 3. Lysine at position 14 is highly conserved, particularly among ERf homologs (> 95%), with occasional substitutions by functionally equivalent methionine or arginine, as seen in *A. thaliana* BRI1, PSKR1, and PepR1. Aspartic acid at position 17 shows ~ 90% conservation, with substitutions by functionally relevant residues like glutamic acid or serine in certain LRR-RLKs (Fig. 2c) (Duszyn et al. 2). BLAST searches using the kinase domain sequence of PpERL1 as the query revealed the presence of homologs in archaea, bacteria, fungi, and algae. Sequence comparison illustrates the progressive formation of the GC motif in RLKs, beginning in prokaryotes, where glycine at position 3 is fully conserved, along with a mosaic distribution of other functional amino acids, continuing through algae, where the first complete motif emerges. Full conservation of the GC motif observed in mosses, with the exception of *Marchantia polymorpha*, has been maintained throughout evolution in vascular plants (Supplementary Fig. 1). This high degree of conservation in the GC motif suggests an essential role of the GC domain in the function of these membrane receptors, highlighting its potential significance in plant signaling mechanisms across diverse evolutionary lineages.

### *In vitro* guanylate cyclase activity of PpERL1

We biosynthesized an N-terminal GST-tagged fragment of PpERL1 (residues 658–1018), including its mutants PpERL1^S877G^ and PpERL1^K890 N^, which contains the kinase and GC domains. The fusion protein predicted in silico molecular weight was approximately 66 kDa. The mass and purity of the purified fusion proteins were analyzed by SDS-PAGE, and the observed protein bands on the gel corresponded well with the predicted molecular weights for each GST-PpERL1 variant (Supplementary Fig. 2).

**Fig. 3:**
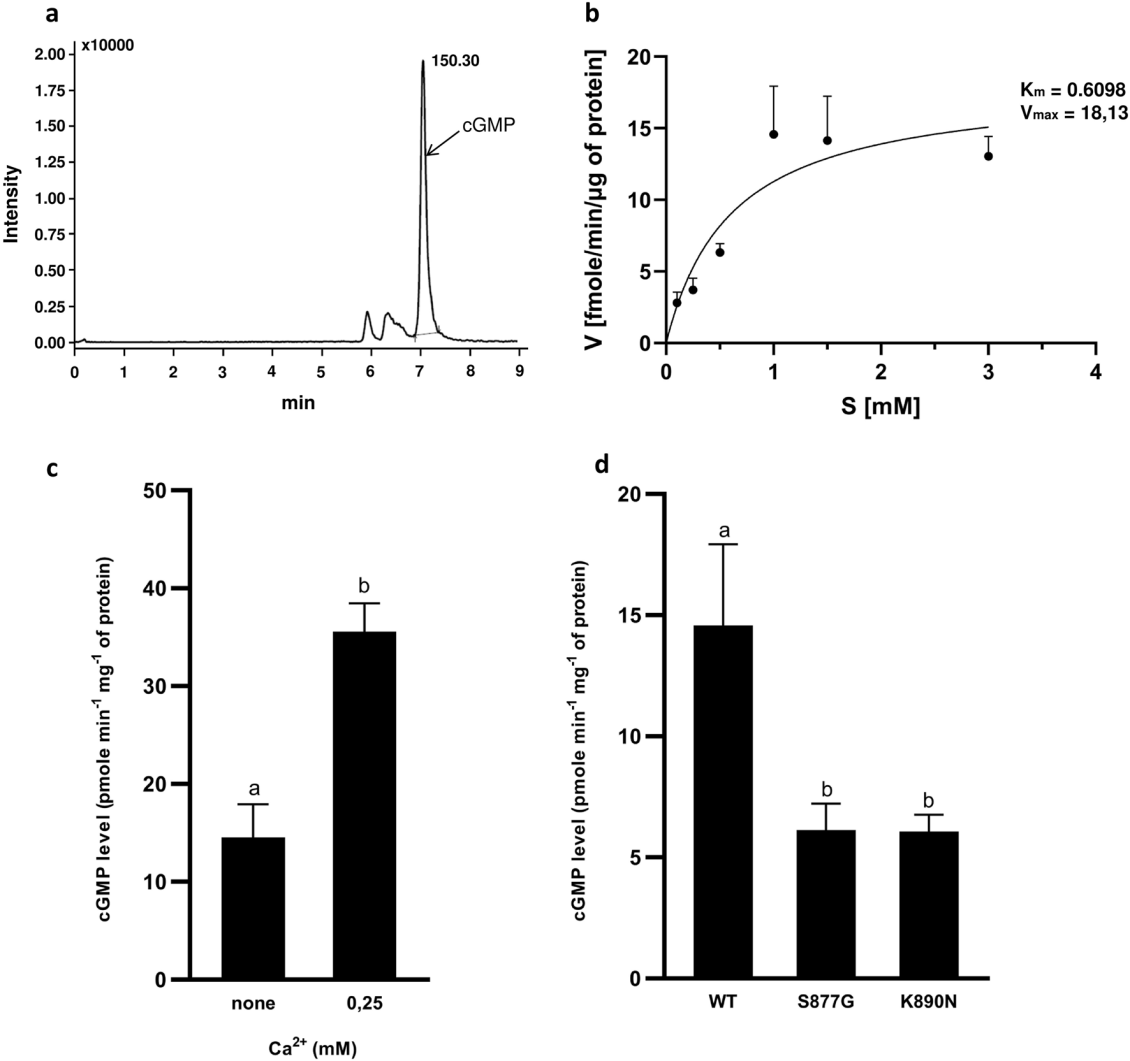
Analysis of guanylate cyclase activity in recombinant PpERL1 proteins, **a** Ion chromatogram of reaction sample with GST-PpERL1 WT and GTP substrate: the chromatogram shows a peak with a retention time of 7.3 min, aligning with the retention time of cGMP; the parent ion is observed at 344.10 m/z, with a daughter ion at 150.30 m/z. **b** GC activity of recombinant PpERL1 WT across GTP concentrations (0.1–3 mM). Data are presented as mean values (n = 3), with error bars representing the standard error of the mean. **c** Effect of Ca^2^⁺ ions on GC activity of PpERL1 WT: reactions were carried out with 1 mM GTP and either with or without 0.25 mM CaCl₂ to assess calcium’s impact on cyclase function. Data are presented as mean values (n = 3), with error bars representing the standard error of the mean. (**d**) cGMP production by PpERL1 WT, K890 N, and S877G mutants: levels of cGMP generated in the presence of 1 mM GTP by PpERL1 WT, PpERL1 K890 N, and PpERL1 S877G. Data are presented as mean values (n = 3), with error bars representing the standard error of the mean

Subsequently, we performed a kinetic analysis to verify the GC activity of the recombinant PpERL1 proteins, demonstrating PpERL1’s ability to convert GTP to cGMP (Fig. 3a) and confirming its active GC domain. Under optimal conditions with 1 mM GTP, the highest GC activity observed was 14.581 pmol mg⁻^1^ min⁻^1^ of cGMP. The enzyme exhibited a K_M_ of 0.6098 mM for GTP and a V_max_ of 18.13 pmol mg⁻^1^ min⁻^1^ (Fig. 3b). This activity level is comparable to that of several known plant GCs; for instance, AtPNP-R1 produces cGMP at 35 pmol mg⁻^1^ min⁻^1^ (Turek and Gehring 26), and HpPepR1 at 17 pmol mg⁻^1^ min⁻^1^ (Świeżawska et al. 22). Other plant GCs, such as AtPSKR1, AtBRI1, AtPepR1 or AtKINγ, have lower activities between 2.5 and 4.5 pmol mg⁻^1^ min⁻^1^ (Kwezi et al. 10, 11; Qi et al. 18; Kwiatkowski et al. 12), while BdPepR2 and the PpERL1 homolog, BdERL1 from *Brachypodium distachyon* show notably higher activities, reaching 112.1 pmol mg⁻^1^ min⁻^1^ and up to 72 pmol mg⁻^1^ min⁻^1^, respectively (Duszyn et al. 2; Świeżawska-Boniecka et al. 23). Further investigation into the effects of calcium ions, inspired by studies suggesting calcium as a molecular switch between kinase and GC activities in LRR-RLKs (Muleya et al. 16), revealed that Ca^2+^ enhanced PpERL1’s GC activity more than twofold, reaching ~ 35.5 pmol mg⁻^1^ min⁻^1^ (Fig. 3c). This response parallels the observed threefold activity increase in AtPSKR1 and a ~ 25% increase in BdERL1 GC activity in the presence of Ca^2+^ (Muleya et al. 16; Świeżawska-Boniecka et al. 23). This evidence thus supports a conserved regulatory role for Ca^2+^ ions in modulating GC domains within LRR-RLKs throughout plant evolution. However, since no distinct Ca^2+^ binding sites have been identified within these receptor structures, the exact regulatory mechanism remains unresolved (Irving et al. 7; Świeżawska-Boniecka et al. 23). To verify the functional integrity of the GC motif in PpERL1, we employed site-directed mutagenesis to alter two critical residues, generating the PpERL1^S877G^ and PpERL1^K890 N^ variants. In the PpERL1^S877G^ mutant, serine (S877) at position 1, essential for guanine binding in GTP, was substituted with glycine, while in PpERL1^K890 N^, lysine (K890) at position 14, involved in stabilizing the cyclization process, was replaced with asparagine. Both mutants displayed approximately 60% reduced GC activity compared to the wild-type protein, underscoring the importance of these residues in maintaining the catalytic pocket’s functional integrity (Fig. 3d). While the significance of the residue at position 1 in the GC catalytic motif remains unreported, lysine at position 14 has been examined in related proteins: analogous mutations at this site result in a twofold reduction of GC activity in BdERL1 (Świeżawska-Boniecka et al. 23) and nearly a fourfold decrease in BdPepR2 (Duszyn et al. 2).

### In vitro analysis of the kinase activity of PpERL1

**Fig. 4:**
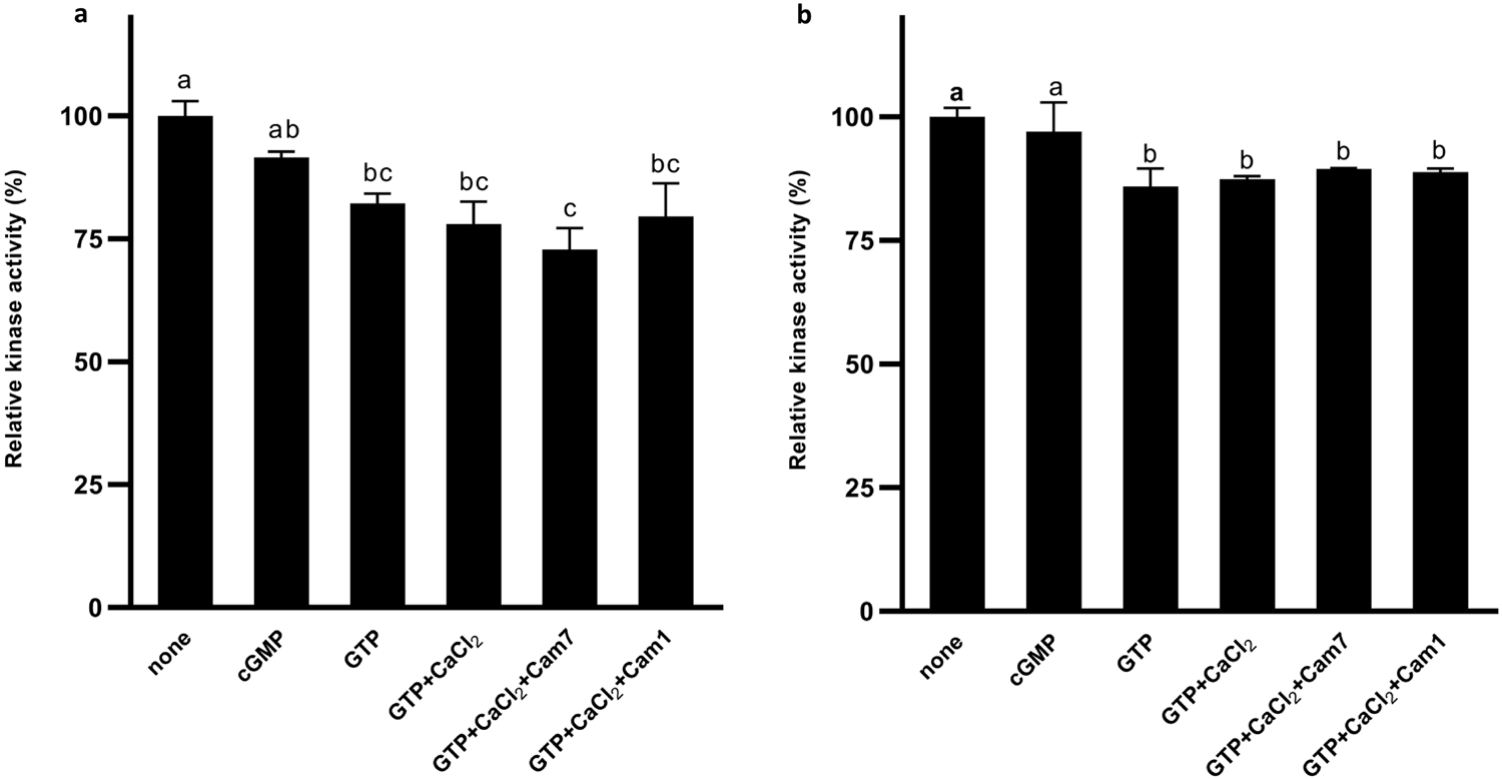
Kinase domain activity of PpERL1 and K890 N mutant proteins**.** **a** Kinase activity of fusion protein PpERL1 WT and **b** Kinase activity of K890 N mutant. Kinase domain activities were assessed under various conditions, including the addition of 2 μM cGMP, 1 mM GTP, 1 mM GTP + 2 μM CaCl₂, and 1 mM GTP + 2 μM CaCl₂ + AtCaM7/AtCaM1 (µg). Data represent mean values (n = 3), with error bars indicating the standard error of the mean

Due to the specific intracellular domains organization of PpERL1 protein, we investigated whether the active GC domain influences on kinase activity by performing phosphorylation reactions in the presence of GTP, cGMP, and GTP with CaCl₂ and/or AtCaM7/AtCaM1 as an intermediate calcium-binding messenger protein (Fig. 4a). Analysis of the PpERL1 protein's kinase domain confirmed its catalytic activity and showed that GTP exerts an inhibitory effect on this activity. Specifically, adding GTP to the reaction mixture led to a significant decrease in kinase activity, consistent with findings in BdPepR2, where GTP reduced kinase activity by half (Duszyn et al. 2). Notably, the presence of calcium ions, which enhance GC activity, did not further inhibit kinase activity beyond the effect of GTP alone. This contrasts with BdERL1 and AtPSKR1, where calcium supplementation led to a substantial or even complete inhibition of kinase activity, suggesting that in PpERL1, calcium acts as a specific regulator for the GC domain only (Muleya et al. 16; Świeżawska-Boniecka et al. 23). Furthermore, kinase domain activity in PpERL1 remained at a control level in the presence of cGMP, indicating that cGMP exerts no significant effect on the kinase activity. This contrasts with findings in BdERL1, BdPepR2, and AtBRI1, where cGMP presence results in an approximate 60% reduction in kinase activity (Wheeler et al. 29; Świeżawska-Boniecka et al. 23; Duszyn et al. 2).

**Fig. 5:**
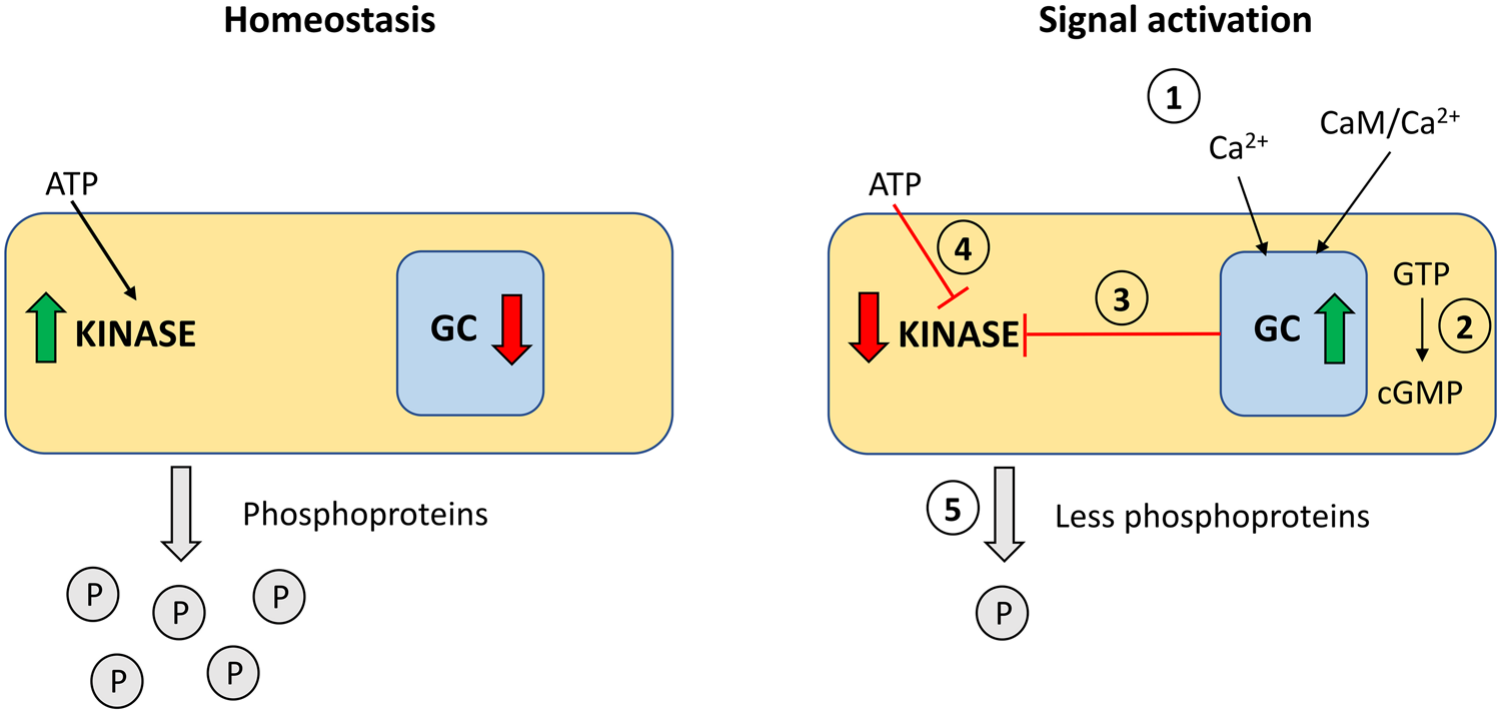
Model of PpERL1 kinase regulation by calcium and the GC domain. During homeostasis, the activity of the GC domain remains dormant or is maintained at a very low level. ATP, which serves as a substrate for the kinase, binds to the ATP-binding site and consequently the kinase can phosphorylate proteins. At the moment of signaling activation (e.g. ion channels), calcium ions appear (**1**) which, when combined with calmodulin, constitute an activation signal for the GC domain. The GTP substrate is converted by guanylate cyclase to cGMP (**2**), which is subsequently used as a particle in further signal transduction. When combined with the presence of the GTP substrate, GC activity can alter the PpERL1 protein's structure (**3**), hence limiting the kinase's activity. These modifications may also physically block the ATP-binding site, preventing ATP from binding (**4**). As a consequence, the availability of the substrate for kinase is reduced and the kinase's capacity to phosphorylate proteins is decreased (**5**)

A comparable response mechanism to that observed in PpERL1 has been identified in the AtKUP5 potassium ion transporter, which contains an AC domain. In this case, although cAMP does not directly stimulate channel activity, a fully functional AC domain is essential for proper K^+^ uptake (Al-Younis et al. 1). The interaction between the kinase domain and the embedded GC domain in PpERL1 diverges from findings in BdERL1, BdPepR1, and AtPSKR1, where it is not the cGMP product of GC activity, but rather the action of the GC domain itself modulates kinase domain activity (Fig. 5). We propose that during the GC activity of PpERL1, the ATP-binding site may experience steric hindrance, which could be attributed to two main factors. First, the substrate of the GC domain could physically obstruct the ATP-binding site, preventing ATP from effectively binding to the kinase domain. This obstruction may occur if the substrate occupies a space that overlaps with the binding site for ATP, thereby impeding its access. Second, conformational changes within the protein structure itself may also play a significant role in limiting substrate access to the kinase domain. Such changes could be induced by the binding of the GC substrate or by the catalytic activity of the GC domain. These alterations in the protein's conformation could create a scenario where the orientation or accessibility of the ATP-binding site is altered, further restricting the interaction with ATP. Together, these mechanisms may serve to modulate the activity of the kinase domain in a way that reflects the dynamic interplay between the GC and kinase functions within PpERL1, ensuring a finely tuned regulatory mechanism that responds to cellular signals and conditions. Given the predicted CaM-binding site in PpERL1, we investigated the potential impact of calmodulin on its kinase activity by testing two calmodulin isoforms from *Arabidopsis thaliana*: CaM1 and CaM7. Our results demonstrated that neither CaM1 nor CaM7 influenced the activity of the kinase domain. This finding is consistent with observations from *Brachypodium distachyon* ERL1 (BdERL1) for CaM1, as well as with *Arabidopsis* BRI1 for CaM6 and PSKR1 for CaM2, all of which underwent in vitro assays (Oh et al. 17; Kaufmann et al. 9; Świeżawska-Boniecka et al. 23). However, pull-down assays indicated that CaM7 and CaM2 could bind to AtBRI1 and AtPSKR1, respectively, in the presence of calcium ions. Notably, this binding occurred primarily with the hypophosphorylated forms of these proteins (Oh et al. 17; Kaufmann et al. 9). Additionally, the coexpression of *AtBRI1* with various calmodulin isoforms inhibited both autophosphorylation and phosphorylation of bacterial proteins. This suggests that the binding of CaM to AtBRI1 in vivo reduces its kinase activity. The authors proposed a regulatory model in which kinase activity is modulated during the initial stages of its activation through the interaction between the Ca^2^⁺/calmodulin complex and the hypophosphorylated form of the kinase. This binding effectively inhibits autophosphorylation, which in turn prevents subsequent transphosphorylation events. By regulating these key phosphorylation processes, the model elucidates how kinase activity influences downstream signaling pathways (Oh et al. 17).

Given the comparable reduction in GC activity observed in both mutants, we selected the K890 N mutant as a representative for further assessment of how the loss of GC domain activity influences the kinase domain's function. We conducted kinase activity assays on the K890 N mutant (Fig. 4b). Our results indicated that cyclic GMP did not significantly impact kinase domain activity. Conversely, the presence of GTP resulted in a slight decrease in kinase activity, though this reduction was less pronounced than that observed in the wild-type PpERL1, suggesting that the GC activity remains partially functional in the mutant. Notably, neither the supplementation with calcium ions nor the introduction of calmodulin isoforms CaM1 or CaM7 further reduced kinase activity beyond what was observed with GTP alone. This finding reinforces the notion that calcium primarily regulates the GC domain rather than directly influencing the kinase domain. The distinct regulatory mechanism governing kinase activity via the GC domain in PpERL1 may stem from structural variations arising from evolutionary divergence, as evidenced by the significantly greater homology observed between monocotyledons and dicotyledons compared to that between mosses and vascular plants.

## Conclusions

Evidence indicates that calcium functions as a conserved regulator of guanylate cyclase activity within leucine-rich repeat receptor-like kinases across plant lineages (Muleya et al. 16; Świeżawska-Boniecka et al. 23), with this mechanism traceable to mosses (Fig. 3c). This suggests Ca^2+^-GC signaling is an ancient, foundational component of plant signaling pathways. The ability of Ca^2+^ to modulate GC and kinase domains may represent a key evolutionary adaptation that enhances signaling flexibility.

Interestingly, in bryophyte LRR-RLKs, kinase activity appears insensitive to both cGMP and Ca^2^⁺ (Fig. 4a) implying alternative regulatory mechanisms may exist in these early-diverging plants, potentially involving specific protein complexes adapted to their environments.

Despite extensive analysis, no cGMP or Ca^2^⁺ binding sites have been identified in LRR-RLKs, yet vascular plants exhibit kinase activity modulation by these molecules in vitro (Muleya et al. 16; Świeżawska-Boniecka et al. 23). This may point to regulation via conformational changes or domain interactions unique to flowering plants.

The complete conservation of a regulatory motif in mosses and some algae supports the idea that LRR-RLK regulation originated in these lineages. Evolutionary divergence likely led to structural and regulatory shifts, particularly in response to cGMP. These findings highlight the complexity of LRR-RLK regulation and underscore the need for further investigation into the molecular mechanisms underlying calcium's role in plant signaling.

## Supplementary Information

Below is the link to the electronic supplementary material.

Supplementary file1 (DOCX 14 KB)

Fig. S1 Evolutionary analysis and sequence comparison of the kinase domains among select groups of organisms. The phylogenetic tree was constructed based on the comparison of kinase domain sequences from selected representatives of different organism groups. The alignments of sequences homologous to the guanylate cyclase motif are labeled according to the ClustalX color scheme, with functional amino acid positions marked by arrows on the identified consensus sequence motif (https://weblogo.berkeley.edu/)

Fig. S2 SDS-PAGE analysis of PpERL1 WT, PpERL1S877G, and PpERL1 K890 N mutants, Lane M – Protein marker: PageRuler™ Plus Prestained Protein Ladder; Lane 1 – GST-tagged PpERL1 WT; Lane 2 – GST-tagged PpERL1S877G; Lane 3 – GST-tagged PpERL1 K890 N

## References

[CR1] Al-Younis I, Wong A, Lemtiri-Chlieh F, Schmöckel S, Tester M, Gehring C, Donaldson L (2018) The arabidopsis thaliana K+-uptake permease 5 (AtKUP5) contains a functional cytosolic adenylate cyclase essential for K+ transport. Front Plant Sci 9:1645. 10.3389/fpls.2018.01645 30483296 10.3389/fpls.2018.01645 PMC6243130

[CR2] Duszyn M, Świeżawska-Boniecka B, Wong A, Jaworski K, Szmidt-Jaworska A (2021) In vitro characterization of guanylyl cyclase BdPepR2 from brachypodium distachyon identified through a motif-based approach. IJMS 22:6243. 10.3390/ijms22126243 34200573 10.3390/ijms22126243 PMC8228174

[CR3] Duszyn M, Świeżawska-Boniecka B, Skorupa M, Jaworski K, Szmidt-Jaworska A (2022) BdGUCD1 and Cyclic GMP Are required for responses of brachypodium distachyon to fusarium pseudograminearum in the mechanism involving jasmonate. IJMS 23:2674. 10.3390/ijms23052674 35269814 10.3390/ijms23052674 PMC8910563

[CR4] Gehring C, Turek IS (2017) Cyclic nucleotide monophosphates and their cyclases in plant signaling. Front Plant Sci 8:1704. 10.3389/fpls.2017.01704 29046682 10.3389/fpls.2017.01704 PMC5632652

[CR5] Hartmann J, Fischer C, Dietrich P, Sauter M (2014) Kinase activity and calmodulin binding are essential for growth signaling by the phytosulfokine receptor PSKR 1. Plant J 78:192–202. 10.1111/tpj.12460 24495073 10.1111/tpj.12460

[CR6] Irving HR, Kwezi L, Wheeler J, Gehring C (2012) Moonlighting kinases with guanylate cyclase activity can tune regulatory signal networks. Plant Signal Behav 7:201–204. 10.4161/psb.18891 22353864 10.4161/psb.18891 PMC3405710

[CR7] Irving HR, Cahill DM, Gehring C (2018) Moonlighting proteins and their role in the control of signaling microenvironments, as exemplified by cGMP and phytosulfokine receptor 1 (PSKR1). Front Plant Sci 9:415. 10.3389/fpls.2018.00415 29643865 10.3389/fpls.2018.00415 PMC5883070

[CR8] Kasahara M, Suetsugu N, Urano Y, Yamamoto C, Ohmori M, Takada Y, Okuda S, Nishiyama T, Sakayama H, Kohchi T, Takahashi F (2016) An adenylyl cyclase with a phosphodiesterase domain in basal plants with a motile sperm system. Sci Rep 6:39232. 10.1038/srep39232 27982074 10.1038/srep39232 PMC5159850

[CR9] Kaufmann C, Motzkus M, Sauter M (2017) Phosphorylation of the phytosulfokine peptide receptor PSKR1 controls receptor activity. J Exp Bot 68:1411–1423. 10.1093/jxb/erx030 28338789 10.1093/jxb/erx030 PMC5441923

[CR10] Kwezi L, Meier S, Mungur L, Ruzvidzo O, Irving H, Gehring C (2007) The arabidopsis thaliana brassinosteroid receptor (AtBRI1) contains a domain that functions as a guanylyl cyclase in vitro. PLoS ONE 2:e449. 10.1371/journal.pone.0000449 17520012 10.1371/journal.pone.0000449 PMC1867859

[CR11] Kwezi L, Ruzvidzo O, Wheeler JI, Govender K, Iacuone S, Thompson PE, Gehring C, Irving HR (2011) The phytosulfokine (PSK) receptor is capable of guanylate cyclase activity and enabling cyclic GMP-dependent signaling in plants. J Biol Chem 286:22580–22588. 10.1074/jbc.M110.168823 21504901 10.1074/jbc.M110.168823 PMC3121402

[CR12] Kwiatkowski M, Wong A, Fiderewicz A, Gehring C, Jaworski K (2024) A SNF1-related protein kinase regulatory subunit functions as a molecular tuner. Phytochemistry 224:114146. 10.1016/j.phytochem.2024.114146 38763313 10.1016/j.phytochem.2024.114146

[CR13] Ludidi N, Gehring C (2003) Identification of a novel protein with guanylyl cyclase activity in arabidopsis thaliana. J Biol Chem 278:6490–6494. 10.1074/jbc.M210983200 12482758 10.1074/jbc.M210983200

[CR14] Malukani KK, Ranjan A, Hota SJ, Patel HK, Sonti RV (2020) Dual activities of receptor-like kinase OsWAKL21.2 induce immune responses. Plant Physiol 183:1345–1363. 10.1104/pp.19.01579 32354878 10.1104/pp.19.01579 PMC7333719

[CR15] Meier S, Ruzvidzo O, Morse M, Donaldson L, Kwezi L, Gehring C (2010) The arabidopsis wall associated kinase-like 10 gene encodes a functional guanylyl cyclase and is co-expressed with pathogen defense related genes. PLoS ONE 5:e8904. 10.1371/journal.pone.0008904 20126659 10.1371/journal.pone.0008904 PMC2811198

[CR16] Muleya V, Wheeler JI, Ruzvidzo O, Freihat L, Manallack DT, Gehring C, Irving HR (2014) Calcium is the switch in the moonlighting dual function of the ligand-activated receptor kinase phytosulfokine receptor 1. Cell Commun Signal 12:60. 10.1186/s12964-014-0060-z 25245092 10.1186/s12964-014-0060-z PMC4180545

[CR17] Oh M-H, Kim HS, Wu X, Clouse SD, Zielinski RE, Huber SC (2012) Calcium/calmodulin inhibition of the *Arabidopsis* BRASSINOSTEROID-INSENSITIVE 1 receptor kinase provides a possible link between calcium and brassinosteroid signalling. Biochemical Journal 443:515–523. 10.1042/BJ20111871 22309147 10.1042/BJ20111871 PMC3316158

[CR18] Qi Z, Verma R, Gehring C, Yamaguchi Y, Zhao Y, Ryan CA, Berkowitz GA (2010) Ca ^2+^ signaling by plant *Arabidopsis thaliana* Pep peptides depends on AtPepR1, a receptor with guanylyl cyclase activity, and cGMP-activated Ca ^2+^ channels. Proc Natl Acad Sci USA 107:21193–21198. 10.1073/pnas.1000191107 21088220 10.1073/pnas.1000191107 PMC3000296

[CR19] Qi L, Kwiatkowski M, Chen H, Hoermayer L, Sinclair S, Zou M, Del Genio CI, Kubeš MF, Napier R, Jaworski K, Friml J (2022) Adenylate cyclase activity of TIR1/AFB auxin receptors in plants. Nature 611:133–138. 10.1038/s41586-022-05369-7 36289340 10.1038/s41586-022-05369-7

[CR20] Shpak ED (2013) Diverse roles of *ERECTA* family genes in plant development. JIPB 55:1238–1250. 10.1111/jipb.12108 24016315 10.1111/jipb.12108

[CR21] Świeżawska B, Jaworski K, Szewczuk P, Pawełek A, Szmidt-Jaworska A (2015) Identification of a Hippeastrum hybridum guanylyl cyclase responsive to wounding and pathogen infection. J Plant Physiol 189:77–86. 10.1016/j.jplph.2015.09.014 26523507 10.1016/j.jplph.2015.09.014

[CR22] Świeżawska B, Jaworski K, Duszyn M, Pawełek A, Szmidt-Jaworska A (2017) The Hippeastrum hybridum PepR1 gene (HpPepR1) encodes a functional guanylyl cyclase and is involved in early response to fungal infection. J Plant Physiol 216:100–107. 10.1016/j.jplph.2017.05.024 28609666 10.1016/j.jplph.2017.05.024

[CR23] Świeżawska-Boniecka B, Duszyn M, Hammer K, Wong A, Szmidt-Jaworska A, Jaworski K (2021) Brachypodium distachyon ERECTA-like1 protein kinase is a functional guanylyl cyclase. FBE. 10.52586/E882 10.52586/E882 34937312

[CR24] Szmidt-Jaworska A, Jaworski K, Pawełek A, Kopcewicz J (2009) Molecular cloning and characterization of a guanylyl cyclase, PnGC-1, involved in light signaling in pharbitis nil. J Plant Growth Regul 28:367–380. 10.1007/s00344-009-9105-8

[CR25] Torii UK (2004) Leucine-rich repeat receptor kinases in plants: structure, function, and signal transduction pathways. Int Rev Cytol 234:1–46. 10.1016/S0074-7696(04)34001-5 15066372 10.1016/S0074-7696(04)34001-5

[CR26] Turek I, Gehring C (2016) The plant natriuretic peptide receptor is a guanylyl cyclase and enables cGMP-dependent signaling. Plant Mol Biol 91:275–286. 10.1007/s11103-016-0465-8 26945740 10.1007/s11103-016-0465-8

[CR27] Turek I, Irving H (2021) Moonlighting proteins shine new light on molecular signaling niches. IJMS 22:1367. 10.3390/ijms22031367 33573037 10.3390/ijms22031367 PMC7866414

[CR28] Vaz Dias F, Serrazina S, Vitorino M, Marchese D, Heilmann I, Godinho M, Rodrigues M, Malhó R (2019) A role for diacylglycerol kinase 4 in signalling crosstalk during *Arabidopsis* pollen tube growth. New Phytol 222:1434–1446. 10.1111/nph.15674 30628082 10.1111/nph.15674

[CR29] Wheeler JI, Wong A, Marondedze C, Groen AJ, Kwezi L, Freihat L, Vyas J, Raji MA, Irving HR, Gehring C (2017) The brassinosteroid receptor BRI 1 can generate cGMP enabling cGMP -dependent downstream signaling. Plant J 91:590–600. 10.1111/tpj.13589 28482142 10.1111/tpj.13589

[CR30] Wong A, Gehring C, Irving HR (2015) Conserved functional motifs and homology modeling to predict hidden moonlighting functional sites. Front Bioeng Biotechnol 3:82. 10.3389/fbioe.2015.00082 26106597 10.3389/fbioe.2015.00082 PMC4460814

[CR31] Wong A, Donaldson L, Portes MT, Eppinger J, Feijó J, Gehring C (2020) The *Arabidopsis* diacylglycerol kinase 4 is involved in nitric oxide-dependent pollen tube guidance and fertilization. Development Dev. 10.1242/dev.183715 10.1242/dev.183715 PMC13110872 32220864

