# Supplementary material for: Guanylate cyclase activity in moss: revisiting the role of ERECTA-like receptors
Source: Physiol Mol Biol Plants. 2025 Jun 4;31(5):813–22. doi: 10.1007/s12298-025-01606-1 (PMC12185792; doi:10.1007/s12298-025-01606-1)
Supplement: Supplementary file 1 — Supplementary file1 (DOCX 14 KB) [file 12298_2025_1606_MOESM1_ESM.docx]

**Supplemental tables**

**Table S1** Oligonucleotides used to synthesize target DNA fragment.

| **Sequence** | **Oligonucleotide sequence (5’-3’)** |
| --- | --- |
| PpERL1_658-1018aa_ | GGATCCCCAGGAATTCAAATGTCCAAAGCACCACAAGGG |
|  | GATGCGGCCGCTCGAGTCACATGTTTCGTGAATTTATC |

**Table S2** Oligonucelotides used to introduce point mutations in PpERL1.

| **Sequence** | **Oligonucleotide sequence (5’-3’)** |
| --- | --- |
| S877G | AGAAGTCTGACGTCTACGGCTTCGGTATTGTGCTG |
|  | CAGCACAATACCGAAGCCGTAGACGTCAGACTTCT |
| K890N | GCTGGAGCTTCTCATGGGAAAAAATGCTGTTGATGATG |
|  | CATCATCAACAGCATTTTTTCCCATGAGAAGCTCCAGC |
