# Supplementary figures and images for: Guanylate cyclase activity in moss: revisiting the role of ERECTA-like receptors
Source: Physiol Mol Biol Plants. 2025 Jun 4;31(5):813–22. doi: 10.1007/s12298-025-01606-1 (PMC12185792; doi:10.1007/s12298-025-01606-1)

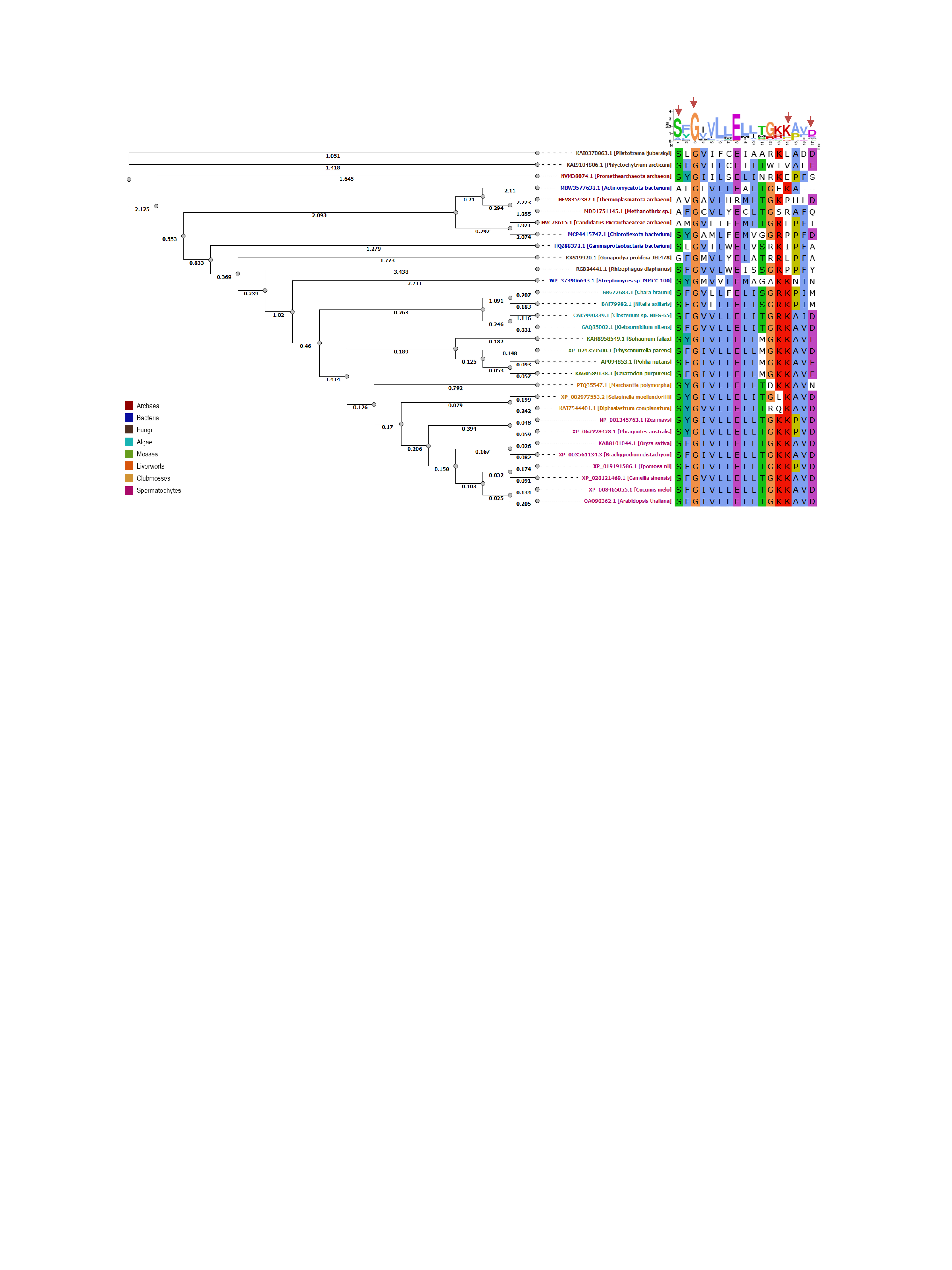

Supplement: Supplementary file 2 — Fig. S1 Evolutionary analysis and sequence comparison of the kinase domains among select groups of organisms. The phylogenetic tree was constructed based on the comparison of kinase domain sequences from selected representatives of different organism groups. The alignments of sequences homologous to the guanylate cyclase motif are labeled according to the ClustalX color scheme, with functional amino acid positions marked by arrows on the identified consensus sequence motif (https://weblogo.berkeley.edu/) [file 12298_2025_1606_MOESM2_ESM.tif]

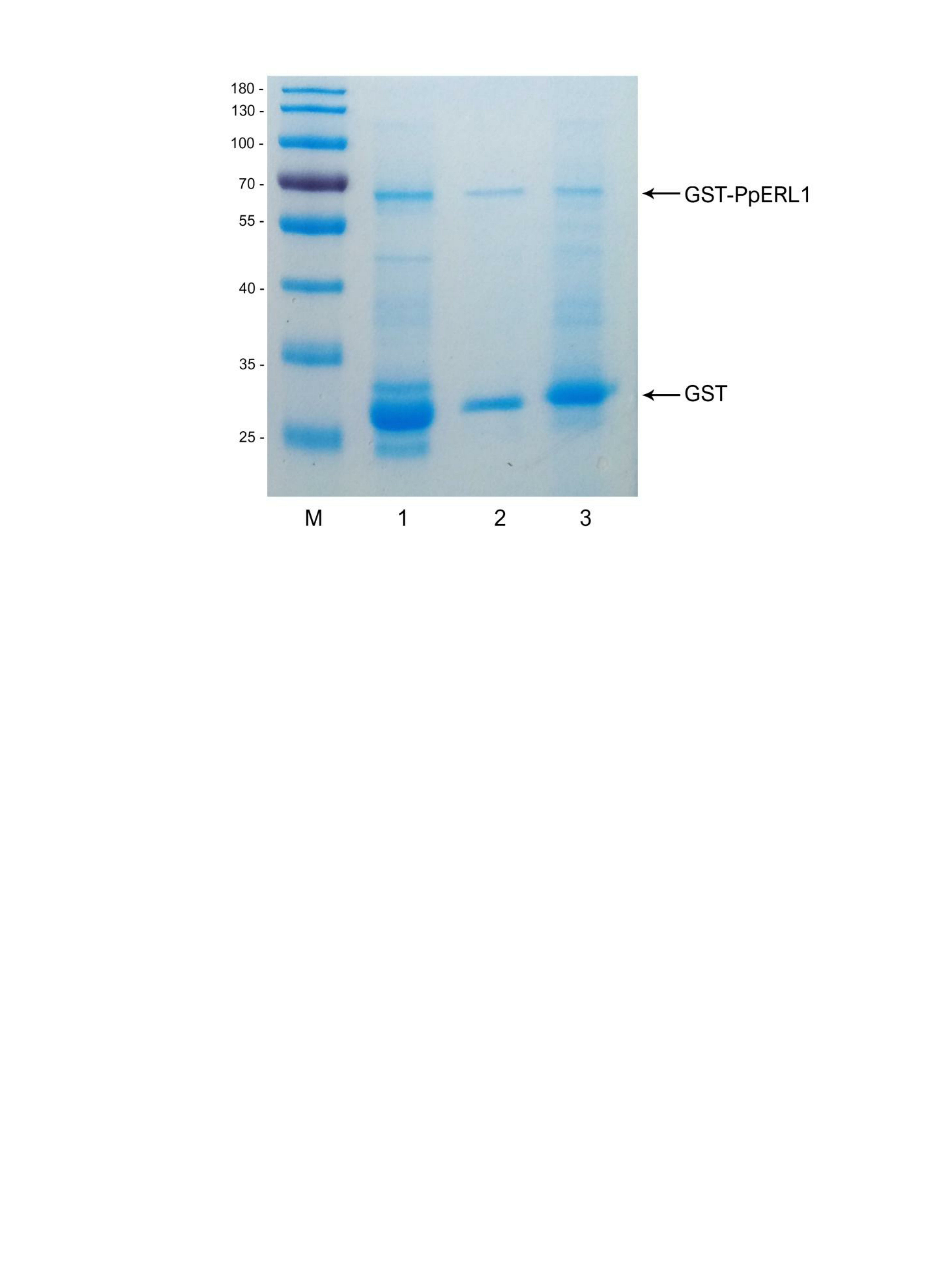

Supplement: Supplementary file 3 — Fig. S2 SDS-PAGE analysis of PpERL1 WT, PpERL1S877G, and PpERL1 K890 N mutants, Lane M – Protein marker: PageRuler™ Plus Prestained Protein Ladder; Lane 1 – GST-tagged PpERL1 WT; Lane 2 – GST-tagged PpERL1S877G; Lane 3 – GST-tagged PpERL1 K890 N [file 12298_2025_1606_MOESM3_ESM.tif]
